# Comparison of corneal morphological characteristics between diabetic and non diabetic population

**DOI:** 10.12669/pjms.336.13628

**Published:** 2017

**Authors:** Qamar Ul Islam, Mohammad Asim Mehboob, Zulfiqar Ali Amin

**Affiliations:** 1 Dr. Qamar Ul Islam, FCPS (Ophthalmology), FCPS (VRO), Department of Ophthalmology, PNS Shifa Hospital, Karachi, Pakistan; 2 Dr. Mohammad Asim Mehboob, MBBS, Department of Ophthalmology, PNS Shifa Hospital, Karachi, Pakistan; 3 Dr. Zulfiqar Ali Amin, FCPS (Med), FCPS (Medical Oncology), Department of Medicine, PNS Shifa Hospital, Karachi, Pakistan

**Keywords:** Corneal Endothelium, Diabetes Mellitus, Endothelial cell density, Specular Microscopy

## Abstract

**Objective:**

To compare corneal morphological parameters between diabetics and age matched non-diabetic control subjects and to evaluate the correlation of these parameters in relation to duration of diabetes mellitus (DM), glycemic status and severity of diabetic retinopathy.(DR).

**Methods:**

This cross sectional comparative study was conducted at the Department of Ophthalmology, PNS Shifa Karachi from February 2016 to January 2017. Patients with ages between 10 to 80 years of either gender who were diagnosed to have DM were recruited in the study. Control group comprised of age matched healthy volunteers who did not have DM. Corneal morphological parameters (CED, Average cell size, CV of cell size and hexagonality) was evaluated in each subject with non-contact specular microscope and findings were endorsed on a pre devised proforma.

**Results:**

Data of 298 eyes (149 diabetic patients and 149 healthy controls) was evaluated. Mean corneal endothelial cell density (CED) of diabetic population was 2494.47 ± 394.10 cells/mm^2^, while mean CED of control group was 2574.46 ± 279.97 cells/mm^2^ [p = 0.04]. Between group differences in mean average cell size, CV of cell size and hexagonality was statistically not significant. Analysis of corneal endothelial parameters among subgroups of patients with no DR, with NPDR and PDR did not show statistically significant difference. Moreover, patients with diabetes of more than 10 years duration had significantly lower CED (p <0.01) and larger average cell size (p= 0.03). Duration of DM was significantly correlated with type of DR, HbA1c level, CED, polymegethism and hexagonality.

**Conclusion:**

Mean corneal endothelial cell density (CED) was found to be significantly lower in diabetic population as compared to healthy controls.

## INTRODUCTION

**Fig.1:**
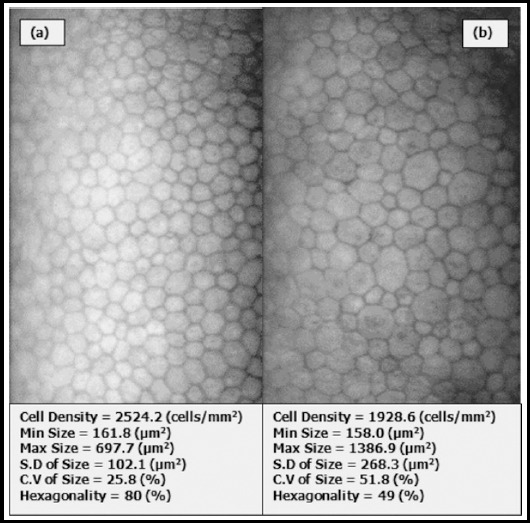
Specular Microscopic photograph. (a) Normal Cornea (b) Diabetic Cornea

Diabetes Mellitus (DM) has become a global epidemic with Pakistan being no exception having 7.0 million patients of DM and the number of diabetic patients is expected to rise to an alarming figure of 14.4 million by the year 2040 making Pakistan the 8^th^ highest country in terms of burden of DM.^1^ Ocular manifestations of DM are manifolds with diabetic retinopathy (DR) being the major complication of DM with significant ocular morbidity. Apart from DR, several structural and functional changes in cornea have been associated with DM that include decrease in corneal endothelial cell density (CED) and hexagonality, increase in central corneal thickness (CCT), polymegethism, pleomorphism, higher corneal auto fluorescence and lower corneal sensitivity^2^,^3^(Fig.1). It is postulated that reduction in the activity of Na^+^- K^+^ ATPase of corneal endothelium in diabetics causes these morphological and functional changes in cornea and consequently damages are caused as corneal compensation against intra ocular pressure (IOP).^3^

Several studies had showed variable results while comparing corneal morphological parameters in diabetics with normal subjects. Lower CED and hexagonality with higher coefficient of variation (CV) and average cell size had been reported by various authors.^3^-^5^ However, there are studies that showed no difference in corneal morphology between diabetics and normal population.^6^-^8^ Correlation of these morphological parameters with duration of DM, type and severity of DR and glycemic control had been studied with variable results. Lee et al. reported patients with DM of > 10 year duration had more corneal morphological abnormalities.^3^ However, Choo et al. found the duration of DM, HbA1c level and severity of DR were not significantly correlated with corneal endothelial findings.^2^ Available data from Pakistan on the subject is limited. Rizvi et al. in their study reported mean CED in Type-2 diabetics being significantly lower than the healthy adults.^9^ In depth analysis of corneal morphological parameters (CED, average cell size, hexagonality and CV) among diabetics and healthy adult population from our country is not available. The objective of this study was to compare corneal morphological parameters (CED, CV, hexagonality and average cell size) between diabetics and age matched non diabetic control subjects and to evaluate the correlation of these parameters in relation to duration of DM, glycemic status and severity of DR.

## METHODS

After approval of hospital ethical review committee, this cross sectional comparative study was conducted at the Department of Ophthalmology, PNS Shifa Naval hospital Karachi from February 2016 to January 2017. Written informed consent was obtained from each subject before enrolment and study was conducted in accordance with the declaration of Helsinki. Patients with ages between 10 to 80 years of either gender who were diagnosed to have DM were recruited in the study through non probability convenience sampling. The diagnosis of diabetes mellitus was based on criteria of the American Diabetes Association (ADA) and included all the patients who were already under treatment of physician.^10^ Control group comprised of age matched healthy volunteers who did not have DM (subjects with fasting blood sugar of less than 110 mg/dL). For detailed analysis DR group was further divided into patients with no DR, non-proliferative DR (NPDR) and those with proliferative DR (PDR) based on the diagnosis by a consultant ophthalmologist. Subjects with refractive error of ≥ ± 1.00 diopters, history of intraocular surgery / trauma /retinal laser, corneal opacity or dystrophy, glaucoma, pseudoexfoliation, uveitis, use of contact lens, use of topical eye drops and diabetes mellitus were excluded. Calculated sample size was 149 in each group keeping level of significance as 0.5, power of test as 90, population mean of CED value as2578 in DR group, and 2605 in control group and population SD as 77.^6^ All the participants underwent complete ocular examination including visual acuity assessment, auto refraction, slit lamp bio microscopic examination of anterior and posterior segment and non-contact IOP measurement. Corneal morphological parameters (CED, Average cell size, CV of cell size and hexagonality) was evaluated in each subject with non-contact specular microscope (SP-3000 P, Topcon Corporation, Japan) by a single experienced examiner between 09:00 -11:00 AM. Three images from central cornea of eye with worse retinopathy stage were captured and 100 contiguous cells per image were included for analysis by built in software. An average of three readings was used for final analysis. All the findings including demographic data, and corneal parameters (CED, mean cell area (MCA), CV of cell size, percentage of hexagonal cells) were endorsed on a pre devised proforma.

Statistical analysis of the data was done using SPSS version 13.0. All the data were tested for normality before analysis. Descriptive statistics i.e. means ± standard deviation (SD) for quantitative variables and frequencies and percentages for qualitative variables were used. Independent sample ‘t’ test and One way analysis of variance (ANOVA) was used to compare quantitative data between groups, while chi square test for independence was used to compare qualitative data. Pearson’s correlation coefficient test was performed to find association of different study variables. A p-value < 0.05 was considered statistically significant.

## RESULTS

Data of 298 eyes (149 diabetic patients and 149 healthy controls) was evaluated. Mean age of diabetic population was 54.13 ± 9.97 years (range: 30-75 years), while mean age of control group was 52.01 ± 12.10 years (range: 32-80 years). Demographic and clinical profile of both groups is given in Table-I. Both groups were matched in terms of age (p=0.10) and gender (p=0.19). Mean fasting plasma glucose level was significantly higher in diabetic group (p <0.01). Mean CED of diabetic population was 2494.47 ± 394.10 cells/mm^2^ (range: 1094.7 – 3358.9 cells/mm^2^), while mean CED of control group was 2574.46 ± 279.97cells/mm^2^ (range: 1856.4 – 3240.6 cells/mm^2^) [p = 0.04]. Between group differences in mean average cell size, CV of cell size and hexagonality was statistically not significant (Table-II). Analysis of corneal endothelial parameters among subgroups of patients with no DR, with NPDR and PDR did not show statistically significant difference (Table-III). However, patients with no DR were significantly younger and had lower HbA1c levels as compared to patients with NPDR and PDR (Table 3). Moreover, patients with diabetes of more than 10 years duration had significantly lower CED (p <0.01) and larger average cell size (p= 0.03). Duration of DM was significantly correlated with type of DR (r = 0.545, p < 0.01), HbA1c level (r =0.165, p = 0.044), CED (r = - 0.282, p <0.01), polymegethism (r = 0.276, p =0.001) and hexagonality (r = 0.162, p = 0.048). Severity of DR showed significant weak correlation with CED (r = - 0.166, p = 0.043) and average cell size (r = 0.185, p = 0.024). Pearson’s correlation analysis showed that plasma glucose and HbA1c levels had no significant correlation with CED, CV, and hexagonality.

**Table-I:** Demographic and clinical profile of study population.

*Parameter*	*Diabetic (n=149)*	*Control (n=149)*	*P-value*
Age (years)	54.13 ± 9.97	52.01 ± 12.10	0.10
Gender			
Male	89 (59.73%)	77 (51.67%)	0.19
Female	60 (40.26%)	72 (48.32%)	
Type Of DM			
Type-1	52 (34.89%)	-	-
Type-2	97 (65.10%)		
Duration of DM			
< 10 years	69 (46.30%)	-	-
> 10 years	80 (53.69%)		
Plasma Glucose (F) mg/dl	180.91 ± 75.67	97.88 ± 12.17	< 0.01
HbA1c Level (%)	6.92 ± 1.26	-	-

**Table-II:** Corneal morphological parameters in diabetics and normal subjects.

*Parameter*	*Diabetic Group (n=149)*		*Control Group(n=149)*		*P-value*

	*Mean ± SD*	*95% CI*	*Mean ± SD*	*95% CI*	
CED (cells/mm^2^) mean ± SD	2494.47 ± 394.10	2430.66 - 2558.27	2574.46 ± 279.97	2,529.13 - 2,619.78	0.044
Avg cell size (µm^2^)mean ± SD	415.31 ± 95.34	399.87 - 430.74	398.99 ± 50.78	390.76 - 407.21	0.066
CV of size (%) mean ± SD	36.03 ± 4.26	35.34 - 36.71	35.86 ± 4.39	35.14 - 36.57	0.739
Hexagonality (%) mean ± SD	52.42 ± 6.94	51.29 - 53.54	52.83 ± 7.44	51.62 - 54.03	0.620

**Table-III:** Corneal Morphological Parameters according to severity of DR.

*Parameter*	*No DR (n=53)*	*NPDR (n=56)*	*PDR (n=40)*	*P-value*
Age (years)	49.66	57.21	55.75	<0.01
Plasma Glucose (mg/dl)	175.66	177.62	192.50	0.525
HbA1c (%)	6.47	7.10	7.28	0.003
CED (cells/mm^2^) mean ± SD	2586.32±331.54	2456.83 ± 384.15	2425.48 ± 465.14	0.099
Avg. cell size (µm^2^) mean ± SD	394.69 ± 56.91	417.61 ± 72.39	439.43 ± 146.98	0.078
CV of size (%) mean ± SD	36.52 ± 4.37	35.22 ± 4.28	36.51 ± 4.01	0.201
Hexagonality (%) mean ± SD	51.36 ± 5.88	53.57 ± 7.78	52.20 ± 6.91	0.245

## DISCUSSION

Hyperglycemia has profound effect on cornea with approximately 70% of diabetics having corneal complications known as diabetic keratopathy.^11^,^12^ Physical instability of corneal endothelium in DM produces a higher susceptibility to surgical stress and other ocular disorders.^13^ Suggested mechanisms of diabetic keratopathy include excessive sorbitol accumulation in corneal endothelium, abnormal patterns of F-actin fibers, and accumulation of advanced glycation end products (AGEs) in the epithelial basement membrane or in Descemet’s membrane.^12^,^13^

Evaluation of corneal morphological parameters i.e. CED, CV and hexagonality has been done worldwide with conflicting reports. Corneal morphological parameters do differ among various races and ethnic groups with age being the major confounding factor affecting the CED, CV and hexagonality. In this study, both groups were age matched to eliminate the age related bias in corneal parameters among groups. In our study, Mean CED of diabetic population was significantly lesser as compared to normal controls (2494.47 cell/mm^2^ vs 2574.46 cells/mm^2^; p=0.04). Significantly lower CED values in diabetic population as compared to healthy controls had been reported in various other studies.^2^,^3^,^14^-^18^ Modis et al.^5^ and Schultz et al.^19^ in their study found significantly lower CED values in Type-I diabetics as compared to controls, whereas in Type-II diabetics the difference was not statistically significant. Roszkowska et al. reported that CED was decreased by 5% in Type-II diabetics and by 11% in Type-I diabetics when compared to healthy subjects.^13^ Batool et al. in their study on Pakistani population found that mean CED in Type-II diabetics was significantly less as compared to healthy adults.^9^

On the contrary, there are studies which documented that diabetic subjects did not differ from non-diabetic controls with regard to CED.^6^,^7^,^8^ Other features of corneal endothelial dysfunction in diabetic patients include polymorphism (decrease in % of hexagonal cells) and polymegethism (increased CV of cell size).^20^ In our study, between groups difference in average cell size, CV of cell size and hexagonality was statistically not significant. Similar results were quoted by various others authors.^6^,^8^,^16^,^18^ However, there are studies that reported significantly more polymegethism and polymorphism in diabetic population as compared to healthy controls.^2^,^3^,^5^,^21^ In our study, severity of DR did not have a significant effect on corneal morphological parameters. Matsuda et al.^21^ and El-Agamy et al.^20^also reported that all diabetic groups (No DR, NPDR and PDR) had no significant difference in endothelial parameters. Whereas, Shukla et al. reported that CV of cell size was higher in patients with PDR.^22^

Correlation between corneal morphological parameters and various systemic and ocular variables such as duration of DM, plasma glucose level, HbA1c level and severity of DR had been extensively evaluated worldwide. In our study, patients with > 10 years of DM had significantly lower CED and larger average cell size. Lee et al.^3^, Briggs et al.^17^and Gupta et al.^23^ reported a significantly higher CV of cell size and lower CED and % of hexagonal cells in diabetics with > 10 years of duration. In our study, duration of DM showed significant correlation with type of DR, HbA1c level, CED, polymegethism and polymorphism. Significant negative correlation between duration of DM and CED had been reported by Modis et al.^5^ and Ashish et al.^15^ in their work. Significant correlation of HbA1c, plasma glucose level and stage of DR with corneal morphological parameters had been reported by Modis et al. in patients with Type-1 DM.^5^ In our study, plasma glucose and HbA1c levels had no significant correlation with CED, CV and hexagonality. Non-significant correlation of duration of DM, HbA1c, glucose level and severity of DR with corneal endothelial parameters had been found in various studies worldwide.^2^,^12^,^14^,^18^,^20^

### Strength and Limitations of the study

The strength of this study was the appropriate sample size, age matched groups, prospective data collection, and evaluation of various corneal parameters (CCT, CV, Avg cell size, and Hexagonality) for the first time in Pakistani population. Limitations of the study include lack of multivariate analysis, not performing gold standard test (glucose tolerance test) to exclude diabetes in controls and not taking into account possible confounding factors like smoking, IOP and corneal diameter. Results of this study provide a greater insight into the understanding of corneal morphology in diabetic population especially in the context of pre-operative evaluation. In fact, Shenoy et al. concluded that evaluation of corneal endothelium in diabetic patients should be part of the protocol for eye care of diabetic patients.^24^

## CONCLUSION

Mean CED was found to be significantly lower in diabetic population as compared to healthy controls. Moreover, duration of DM was significantly correlated with type of DR, HbA1c level, CED, polymegethism and hexagonality.

### Authors’ Contribution

**QUI:** The conception and design of the study, acquisition of data, analysis and interpretation of data, drafting the article and final approval.

**MAM**: Acquisition of data and final approval of manuscript.
